# Transcriptomic profiling reveals RetS-mediated regulation of type VI secretion system and host cell responses in *Pseudomonas aeruginosa* infections

**DOI:** 10.3389/fcimb.2025.1582339

**Published:** 2025-06-10

**Authors:** Yinglin Wu, Shan Huang, Kai Zhang, Yangcheng Shen, Shebin Zhang, Haining Xia, Jieying Pu, Cong Shen, Cha Chen, Jianming Zeng

**Affiliations:** ^1^ The Second Clinical Medical College, Guangzhou University of Chinese Medicine, State Key Laboratory of Traditional Chinese Medicine Syndrome, Guangdong Provincial Hospital of Chinese Medicine, Guangzhou, China; ^2^ Department of Laboratory Medicine, The Second Affiliated Hospital of Guangzhou University of Chinese Medicine, Guangzhou, China; ^3^ Guangdong Provincial Key Laboratory of Research on Emergency in Traditional Chinese Medicine (TCM), The Second Affiliated Hospital of Guangzhou University of Chinese Medicine, Guangzhou, China; ^4^ School of Laboratory Medicine, Guangzhou Health Science College, Guangzhou, China

**Keywords:** *Pseudomonas aeruginosa*, virulence, type VI secretion system, chaperone-usher pathway, CupC fimbriae, A549 epithelial cells, PDE4C

## Abstract

*Pseudomonas aeruginosa* is a major opportunistic pathogen that causes chronic infections, particularly in patients with cystic fibrosis and chronic obstructive pulmonary disease (COPD). The type VI secretion system (T6SS) is a primary virulence factor of *P. aeruginosa* in chronic infections. The objective of this study was to elucidate the regulatory mechanisms and pathogenic effects of the T6SS during *P. aeruginosa* infection, utilizing transcriptome sequencing and functional assays. We found that T6SS expression is elevated in *P. aeruginosa* isolated from chronically infected patients. Deletion of the *retS* gene activates *P. aeruginosa* PAO1 T6SS while repressing T3SS *in vitro*. Bacterial and cellular transcriptome sequencing analyses showed that T6SS genes were upregulated, while T3SS genes were downregulated in the Δ*retS* mutant. Additionally, the expression levels of the fimbriae gene *cupC*, the histidine phosphotransfer protein *hptC* (*PA0033*), and the transcription factor *PA0034* were significantly increased. Subsequent experiments revealed that adhesion mediated by *cupC* enhances the contact-killing activity of the T6SS. Deletion of the *hptC*-*PA0034* operon results in the down-regulation of *cupC* expression. The Δ*retS*Δ*cupC* and Δ*retS*Δ*hptC*-*PA0034* mutants exhibited reduced cytotoxicity compared to the Δ*retS* mutant, similar to the Δ*retS*Δ*clpV1*Δ*clpV2* mutant. The Δ*retS* infection increased cell death, inflammatory factors (IL-1β, IL-6, TNF-α), and reactive oxygen species compared to a T6SS-inactive strain. Importantly, our study demonstrates that the T6SS activates the PDE4C pathway in epithelial cells, leading to significant cellular alterations. The application of PDE inhibitors effectively mitigates cell damage and inflammatory responses. These findings highlight the critical role of T6SS in modulating host cell signaling and suggest potential therapeutic strategies for conditions associated with T6SS-mediated inflammation.

## 1 Introduction

*Pseudomonas aeruginosa* (*P. aeruginosa*) is an extremely adaptable opportunistic pathogen that poses a major threat in clinical settings and can cause acute to chronic opportunistic infections (Luyt et al., 2018; Valentini et al., 2018; Azam and Khan, 2019; Feng et al., 2019; Yin et al., 2021). Especially as one of the most important pathogenic microorganisms of lower respiratory tract chronic infection or wound infection (Garcia-Vidal et al., 2009). The pathological changes of the lesions create a favorable environment for *P. aeruginosa* colonization and chronic infection (Folkesson et al., 2012). *P. aeruginosa* possesses a diverse array of virulence factors that are intricately regulated by intracellular systems to facilitate its pathogenicity (Finch et al., 2015).

One of the key virulence of *P. aeruginosa* is the type VI Secretory System (T6SS), which comprises three distinct clusters: H1-T6SS, H2-T6SS, and H3-T6SS (Sana et al., 2013). This system is a contractile, phage-like nanomachine that delivers toxins into target cells. T6SS-associated effector molecules are diverse, each effector molecule interacts with cognate immune proteins to prevent host cell poisoning and protect the bacterial cells from self-damage (Filloux et al., 2008; Ho et al., 2014; Basler, 2015). The effectors TseT (Burkinshaw et al., 2018), TseV and PAAR2 (Wang et al., 2021b) display antibacterial activity. The Azu protein secreted by the H2-T6SS enhances bacterial competitiveness under low copper ion concentrations (Dutta et al., 2019). TseF facilitates the delivery of outer membrane vesicles (OMV) associated iron to bacterial cells by engaging the Fe(III)-pyochelin receptor FptA and the porin OprF (Lin et al., 2017). The effects of the T6SS of *P. aeruginosa* on eukaryotic cells have been extensively investigated. For instance, PldA has been shown to induce activation of the phosphoinositide 3-kinase (PI3K) pathway. PldB promotes bacterial internalization into epithelial cells by activating the PI3K/Akt signaling pathway. Additionally, VgrG2B and TepB, which are also secreted by the T6SS of *P. aeruginosa* targeting eukaryotic cells, have been implicated in various cellular interactions. For more detailed information, see reference (Monjaras Feria and Valvano, 2020; Yang et al., 2022). A transcriptomic analysis of *P. aeruginosa* isolated from chronic wounds revealed that the T6SS was significantly upregulated in these clinical settings (Kim et al., 2024).

The previous studies found that the T6SS are regulated by RetS (Brencic et al., 2009; Jimenez et al., 2012; Han et al., 2019; Allsopp et al., 2022), a hybrid sensor kinase that directly binds to the sensor kinase GacS to inhibit its phosphorylation, switching the T3SS and T6SS of *P. aeruginosa* (Goodman et al., 2009; Sonnleitner et al., 2012). The effects of T6SS on pathogenesis and bacterial competition are well established *in vitro*, but its functions and mechanisms *in vivo* are less well understood. To investigate the functional role of T6SS, we constructed a *retS* knockout strain of *P. aeruginosa* PAO1, which exhibits enhanced T6SS expression and suppressed T3SS activity. Transcriptome sequencing and functional assays were subsequently employed to explore the regulatory mechanisms and pathogenic effects of T6SS during *P. aeruginosa* infection.

## 2 Materials and methods

### 2.1 Bacterial strains and growth conditions

The *P. aeruginosa* PAO1 strain was provided by Professor Zhou from the Children’s Hospital of Chongqing Medical University. Bacterial cultures were grown in fresh Luria-Bertani (LB) medium or on LB agar plates (15% agar) at 37°C. When required, antibiotics were added to the medium or agar plates at the following concentrations: 30 µg/mL gentamicin and 16 µg/mL chloramphenicol.

### 2.2 Construction of *P. aeruginosa* deletion mutants

In this study, we constructed gene deletion mutants of *P. aeruginosa* PAO1, targeting the *retS*, *cupC* (*cupC1-cupC3*), *clpV1, clpV2 and PA0033*-*PA0034* loci. The deletions were achieved using a *sacB*-based suicide vector system, following a previously described method (Lu et al., 2019). Briefly, the upstream and downstream flanking sequences of *retS* were fused by PCR and inserted into the *XbaI*/*SacI* sites of pGSM to create pGSM-*retS*. This plasmid was then transformed into PAO1 to generate the *retS* mutant. Based on the *retS* mutant, we sequentially constructed pGSM-*clpV1* and pGSM-*clpV2* plasmids and transformed them into Δ*retS* to obtain the triple mutant Δ*retS*Δ*clpV1*Δ*clpV2*. Similarly, pGSM-*cupC* and pGSM-*PA0033–34* plasmids were constructed and transformed into Δ*retS* to generate the Δ*retS*Δ*cupC* and Δ*retS*Δ*hptC*-*PA0034* mutants. Detailed information on the bacterial strains, plasmids, and primers used in this study is provided in **Supplementary Tables S1**, **S2**.

### 2.3 Bacterial competition assay

Predator strains (PAO1 and mutant-derived strains) and prey strains (*E. coli* DH5α, gentamicin resistance) were grown to the mid-exponential phase. The cultures were collected, resuspended in PBS to achieve an optical density (OD) of 1.0 at 600 nanometers (nm), and mixed at a 1:1 ratio. A volume of 25 μL of the mixed bacterial culture was spotted onto pre-warmed LB agar plates and incubated at 37°C for 24 h. Bacterial spots were harvested, and the recovered cells were inoculated into a selective medium containing 30 µg/mL gentamicin. The viability of prey cells was then measured (Lin et al., 2015).

### 2.4 Pyocyanin production assay

Pyocyanin was extracted from 5 mL of *P. aeruginosa* culture supernatant using 3 mL of chloroform and 1 mL of 0.2 N HCl. The absorbance of the extract was measured at 520 nm and 600 nm, and the pyocyanin concentration was calculated using the formula (Essar et al., 1990): (OD_520_/OD_600_ × 17.072) = μg/mL. All experiments were conducted independently and in triplicate.

### 2.5 Biofilm formation assay

A biofilm formation assay was performed as previously described by Sara Carloni (Carloni et al., 2017). Mid-exponential phase bacteria were inoculated into 2 mL of LB broth at an OD_600_ of 0.05 in 12-well plates and incubated for 24 h at 37°C. Biofilms were stained with 1% crystal violet, and the attached cells were solubilized with 2 mL of 95% ethanol. The absorbance of the solubilized crystal violet was measured at OD_600_. All experiments were conducted independently in triplicate.

### 2.6 Survival curve of *Galleria mellonella* larvae

Bacteria were cultured and collected according to the method described above. After washing and resuspending the bacteria with PBS, the concentration of the bacterial solution was adjusted to 2×10^3^ colony-forming units (CFU)/mL. Phosphate-buffered saline (PBS) was used as a negative control. In the experimental group, 10 μL of bacterial solution (containing 60 CFU) was injected into each larva, and each group had 10 larvae. The survival of wax moth larvae was recorded after incubation at 37°C. The survival curve of wax moth larvae was drawn based on the survival time and survival rate. All procedures were performed in a biosafety cabinet following 30 minutes (min) of ultraviolet irradiation. All experiments were conducted independently in triplicate.

### 2.7 Cell culture and infection

The A549 cell line (ATCC: CCL-185), derived from human pulmonary epithelial cells, was maintained in Dulbecco’s Modified Eagle’s Medium (DMEM) supplemented with 10% fetal bovine serum (FBS; Gibco, Carlsbad, CA). The THP-1 cell line (ATCC: CL-0233), derived from human acute monocytic leukemia cells, was maintained in RPMI 1640 medium (Gibco) supplemented with 10% FBS and 1% penicillin/streptomycin (P/S). Both cell lines were cultured at 37°C in a 5% CO_2_ atmosphere and passaged at a 1:5 ratio every 3 days.

For experiments, A549 cells were seeded in well plates and incubated at 37°C for 14 hours (h). THP-1 cells were treated with PMA (phorbol 12-myristate 13-acetate) for 24 h to induce differentiation and adherence to the culture surface. Bacterial strains were grown to logarithmic phase, harvested, and resuspended in PBS. Before infection, the cell culture medium was replaced with DMEM or RPMI 1640 containing 1% FBS. Infection doses were determined based on bacterial suspension absorbance at 600 nm (OD_600_). Bacterial CFU were calculated using the formula: CFU (10^8^ CFU/mL) = 22.031 × OD_600_ + 0.8278.

### 2.8 Cytotoxicity assay

Cytotoxicity assays quantify cellular damage by measuring lactate dehydrogenase (LDH) activity released into the culture medium using the LDH Cytotoxicity Assay kit (Beyotime Biotechnology, Shanghai, China). A549 cells were seeded at a density of 4 × 10^5^ cells per well in a 12-well plate one day prior to infection. Cells were infected with specified bacterial strains at a multiplicity of infection (MOI) of 50 and maintained at 37°C with 5% CO_2_ for 12 h. Three control groups were included: wells with cells infected with bacteria (co-culture), cell-free culture wells (DMEM control), and uninfected cells (cell control). For the LDH assay, the maximum lysis control well (positive control) was treated with LDH release reagent for 1 hour, and samples were processed according to the manufacturer’s instructions Absorbance readings were taken at 490 nm (OD_490_) and 900 nm (OD_900_) simultaneously, with OD_900_ subtracted from OD_490_ to correct for background. The percentage of cytotoxicity was calculated using the formula: (A_co-culture_ – A_cell control_)/(A_positive control_ – A_cell control_) × 100%. All experiments were conducted independently in triplicate.

### 2.9 Calcein-AM/PI live-dead cell staining

The A549 cells (2 × 10^5^ cells/well) were plated in 24-well plates and infected with PAO1 strains at an MOI of 50, while the control group received 1% FBS in DMEM. After 7 h of infection, the supernatant was discarded, and cells were rinsed with PBS. A working solution was prepared by mixing 2 μL Calcein AM (1 mM) and 2 μL propidium iodide (PI, 2 mM). 200 μL of this solution was added to each well, and cells were incubated at 37°C for 30 min. Live cells were stained green, and dead cells were stained red, observed using a fluorescence inverted microscope. All experiments were conducted independently in triplicate.

### 2.10 Intracellular ROS generation assay

PMA-differentiated THP-1 cells were treated as indicated. After bacterial infection, a fresh medium containing 10 μM DCFH-DA (Sigma-Aldrich) was added to the cells for 30 min. 0.05mg/ml Rosup was added to the positive control wells to induce ROS production. The cells were then washed three times with PBS, and the fluorescence intensity before and after stimulation was measured using a fluorescence spectrophotometer with an excitation wavelength of 488 nm and an emission wavelength of 525 nm. All experiments were conducted independently in triplicate.

### 2.11 CCK-8 assay

A549 cells were seeded in a 96-well plate at a density of 5×10^4^ cells per well in 100 μL of culture medium. After 12–16 h of culture, the experimental groups (As) were treated with the PDE4 inhibitor Rolipram at concentrations of 0 nM, 3 nM, 30 nM, and 300 nM. Each treatment condition was performed in triplicate. Additionally, a blank well (Ac) containing only culture medium without cells and a control well (Ab) containing untreated cells with culture medium were included. After treatment, the culture supernatant was discarded, and 200 μL of fresh culture medium was added to each well, followed by the addition of 10 μL of CCK-8 reagent. The OD_450_ was measured using a microplate reader following 2 h incubation period. The cell viability was calculated using the following formula: [(Ac−Ab)/(As−Ab)] × 100%.

### 2.12 RNA-seq

A549 cells (10×10^5^ cells/well) were inoculated in 6-well plates and infected with PAO1 and Δ*retS* at an MOI of 10 for 12 h. Following infection, bacterial cells in the supernatant were collected by centrifugation at 8,000 × g for 10 min at 4°C. Host cells adhering to the culture plate were lysed directly using TRIzol reagent. Subsequent transcriptome sequencing was performed by Shanghai Meiji biological medical technology Co Ltd. Two independent biological replicates were conducted.

### 2.13 Data analysis

The low-quality reads (PHRED score < 20 and read length < 25 bp) were trimmed and filtered out using fastp (v0.21.0) (Chen et al., 2018). The sequencing reads obtained from A549 cells were mapped to the *Homo sapiens* reference genome GRCh38.p13 ( *http://ftp.ensembl.org/pub/release-105/fasta/homo_sapiens/dna/Homo_sapiens.GRCh38.dna.primary_assembly.fa.gz* ). Similarly, the processed and cleaned reads derived from *P. aeruginosa* were aligned to the *P. aeruginosa* strain PAO1 reference genome, identified by the GenBank accession number NC_002516.2. The alignment procedures were executed utilizing the HISAT2 (v2.1.0) (Kim et al., 2019). All files in the SAM format were converted to BAM format and sorted by samtools (v1.18) (Danecek et al., 2021). The sorted BAM files and annotation files were fed into StringTie (v2.1.4) for the estimation of transcript abundance (Pertea et al., 2015). The Python script prepDE.py, which is included with StringTie software, was employed to directly extract read count information from the output files generated by StringTie. Lastly, the differential expression analysis was leveraged by the R package DESeq2 (v1.38.3) within the RStudio console (R v4.2.3, RStudio v2021.09.0). To conducting Kyoto Encyclopedia of Genes and Genomes (KEGG) and Gene Ontology (GO) enrichment analyses, we employed lists of differentially expressed genes that met the following stringent criteria: a False Discovery Rate (FDR) less than 0.01, and an absolute value of log2(Fold Change) exceeding 1.5. The KEGG Pathways and GO terms enrichment analysis were performed with R package ClusterProfiler (v4.2.2) (Wu et al., 2021). GO terms and KEGG Pathways with FDR ≤ 0.05 were screened for significant enrichment.

### 2.14 ncRNA prediction

ncRNA and antisense RNA were identified by using Rockhopper software (Tjaden, 2020), which takes RNA sequencing reads (*.fastq) as input file. The genomic locations of the predicted ncRNAs and antisense RNAs, including their start and end sites as well as strand information, were compiled in *.gff format and subsequently integrated into the *.gff file of PAO1. The enhanced *.gff file was utilized for gene quantification by StringTie and subsequent differential gene expression analysis using DESeq2.

### 2.15 qRT-PCR

Bacteria and cells were harvested by centrifugation and subsequently lysed using TRIzol Reagent (Takara Bio Inc.). Total RNA extraction followed the manufacturer’s protocol and RNA quantity was assessed using a Nanotrap 2000 spectrophotometer. For reverse transcription, 1 µg of RNA was converted to cDNA using the PrimeScript RT reagent kit (TaKaRa, Dalian, China). The resulting cDNA samples were analyzed via quantitative Reverse Transcription Polymerase Chain Reaction (qRT-PCR) on ViiATM 7 Dx system (Applied Biosystems, Foster, CA, USA) using SYBR Green Premix Pro Taq HS qPCR Kit (Accurate Biology, Changsha, China). To determine gene expression levels, qRT-PCR data were normalized against reference genes, *rpoD for P. aeruginosa* strains or *β-actin* for A549 cells, and analyzed using the comparative threshold cycle method (2^-ΔΔCt^). Specific primer sequences used are detailed in **Supplementary Table S1**. All experiments were conducted independently in triplicate.

### 2.16 cAMP assay

The production of cAMP in cell supernatants was measured using an ELISA kit (Elabscience) according to the manufacturer’s instructions. All experiments were conducted independently in triplicate.

### 2.17 Statistical analysis

Data of the results from multiple independent experiments are expressed as the means ± standard deviation (SD). The differences between groups were analyzed using Student’s *t*-test when two groups were compared or one-way ANOVA when more than two groups were compared. All analyses were performed using GraphPad Prism, version 5 (GraphPad Software, Inc., San Diego, CA, USA). Differences with a *P*-value (*P*) of 0.01  <  *P* < 0.05 are represented by *, *P  *<  0.01 are represented by **, and *P*  <  0.001 are represented by ***.

## 3 Result

### 3.1 The T6SS of *P. aeruginosa* is activated during chronic infections

**Figure 1:**
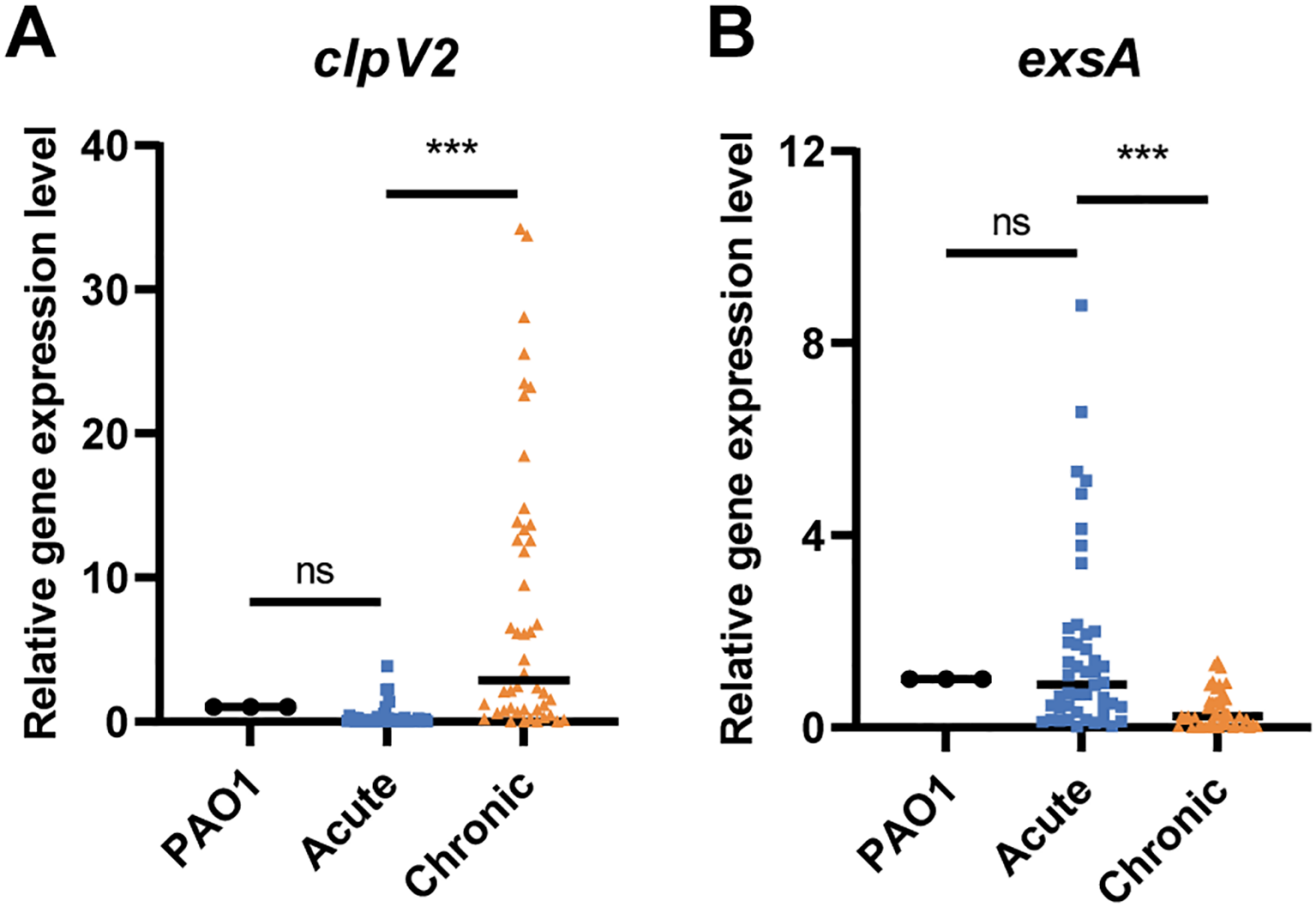
The expression of **(A)** *clpV2* and **(B)** *exsA* in *P. aeruginosa* isolates from acute and chronic respiratory infections. The acute and chronic groups included 46 strains, respectively. ns, not significant. ***P  <  0.001.

To elucidate the activation profiles of T3SS and T6SS in acute and chronic respiratory infections, we analyzed a cohort comprising 46 isolates from patients with chronic *P. aeruginosa* infections, as previously described (Shen et al., 2023), and 46 strains isolated from patients with acute *P. aeruginosa* infections. qRT-PCR analysis showed that the expression of the T6SS gene *clpV2* was significantly higher in the chronic infection group than in the acute infection group (*P* < 0.001), while the expression of the T3SS master regulator-encoding gene *exsA* was downregulated (*P* < 0.001) (**Figure 1**). These observations suggest that T6SS plays a predominant role in the chronic infection process, consistent with findings reported in the literature.

### 3.2 Knocking out *retS* reduced *P. aeruginosa* virulence by activating T6SS and inhibiting T3SS

**Figure 2:**
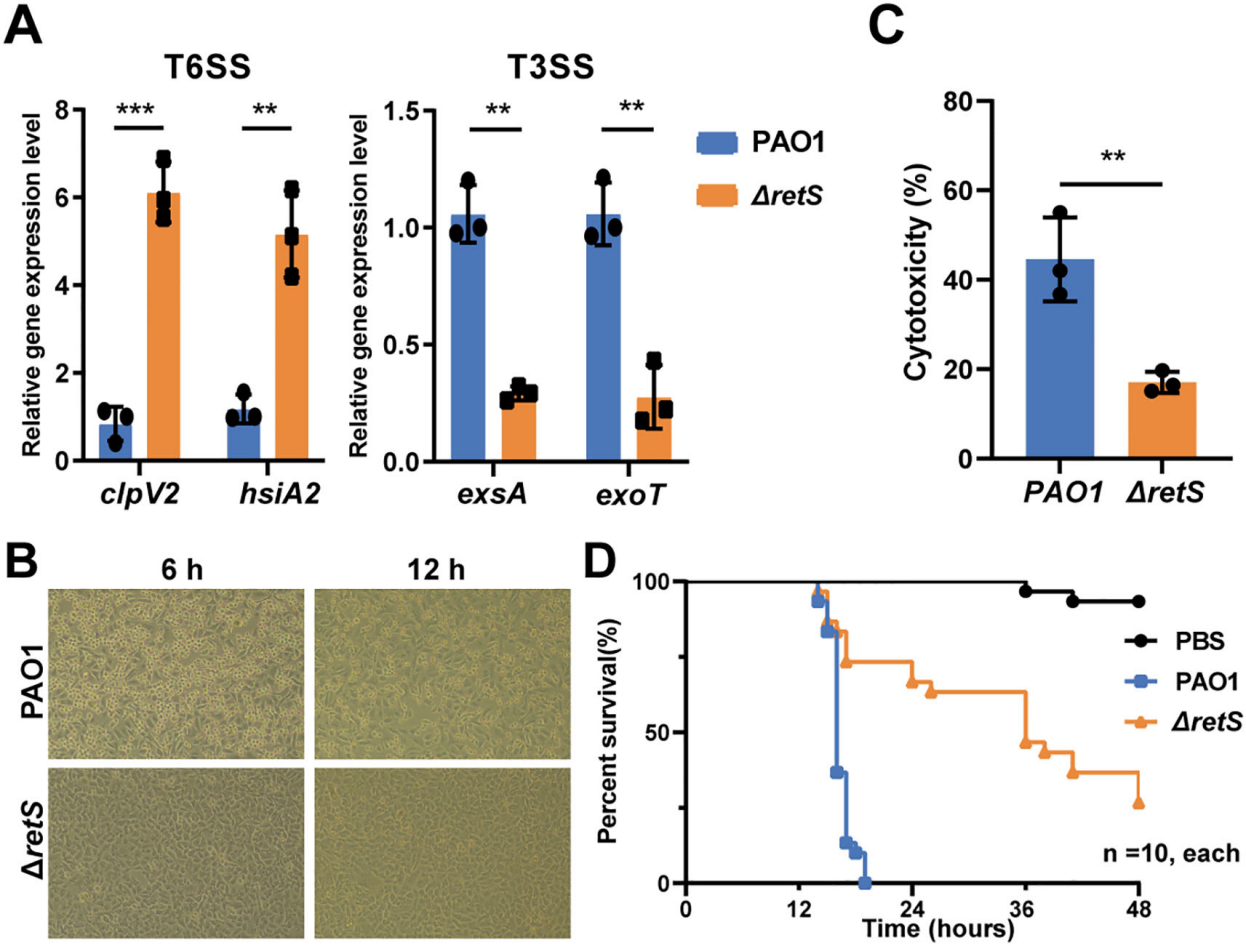
Knocking out *retS* gene reduces the virulence of *P. aeruginosa* by activating T6SS and inhibiting T3SS. **(A)** qRT-PCR detection of the expression levels of T6SS genes *clpV1* and *hsiA2*, and T3SS genes *exsA* and *exoT* in the *P. aeruginosa* PAO1 WT and Δ*retS* strains. **(B)** A549 human lung epithelial cells were infected with PAO1 WT and Δ*retS* strains, MOI=10. **(C)** LDH release assay of A549 cells infected with PAO1 WT and Δ*retS* strains at an MOI of 10 for 12 h. **(D)** Kaplan-Meier survival curve of *G*. *mellonella* larvae infected with PAO1 WT and Δ*retS* strains at a dose of 60 CFU per worm, 10 in each group. **P  <  0.01, ***P  < 0.001, n=3.

Studies have shown that the three clusters of T6SS (H1, H2, and H3) in *P. aeruginosa* are regulated by RetS (Brencic et al., 2009). RetS controls the switch between T3SS and T6SS in *P. aeruginosa* (Goodman et al., 2009; Sonnleitner et al., 2012; Wang et al., 2021a). In the *retS* knockout strain, the expression levels of the T6SS representative genes *clpV2* and *hsiA2* were significantly upregulated compared with the *P. aeruginosa* PAO1 wild-type (WT) strain (*P* < 0.001 and *P* < 0.01, respectively). Conversely, the expression levels of the T3SS representative genes *exsA* and *exoT* were significantly downregulated (*P* < 0.01 for both) (**Figure 2A**). Additionally, the bacterial competition assay revealed that the Δ*retS* strain exhibited significantly enhanced competitive fitness against *E. coli* compared with the PAO1 WT strain (**Supplementary Figure S1C**). Furthermore, both biofilm formation and pyocyanin production were significantly increased in the Δ*retS* strain (**Supplementary Figures S1D, E**).

Subsequently, the virulence capabilities of the strains were compared using a cell infection model. Morphological observations of A549 cells infected with the PAO1 WT strain revealed a rounded and detached cell morphology at both 6 h and 12 h post-infection, in contrast to those infected with the Δ*retS* strain (**Figure 2B**). Additionally, at 12 h post-infection, the level of lactate dehydrogenase (LDH) release in the Δ*retS*-infected group was significantly lower than that in the PAO1 WT strain infected group (*P* < 0.01) (**Figure 2C**). Additionally, the results from the *Galleria mellonella* larvae infection model demonstrated that the mortality rate of the Δ*retS*-infected group was significantly lower than that of the PAO1 WT strain infected group (*P* < 0.001) (**Figure 2D**). Collectively, these results indicate that the virulence of the *retS* mutant is attenuated compared to the PAO1 WT strain. This attenuation may be related to the down-regulation of key T3SS genes following deletion of the *retS* gene, which in turn reduces acute damage to host cells.

### 3.3 Transcriptomic profiling reveals RetS-mediated reprogramming of virulence and host cell responses

**Figure 3:**
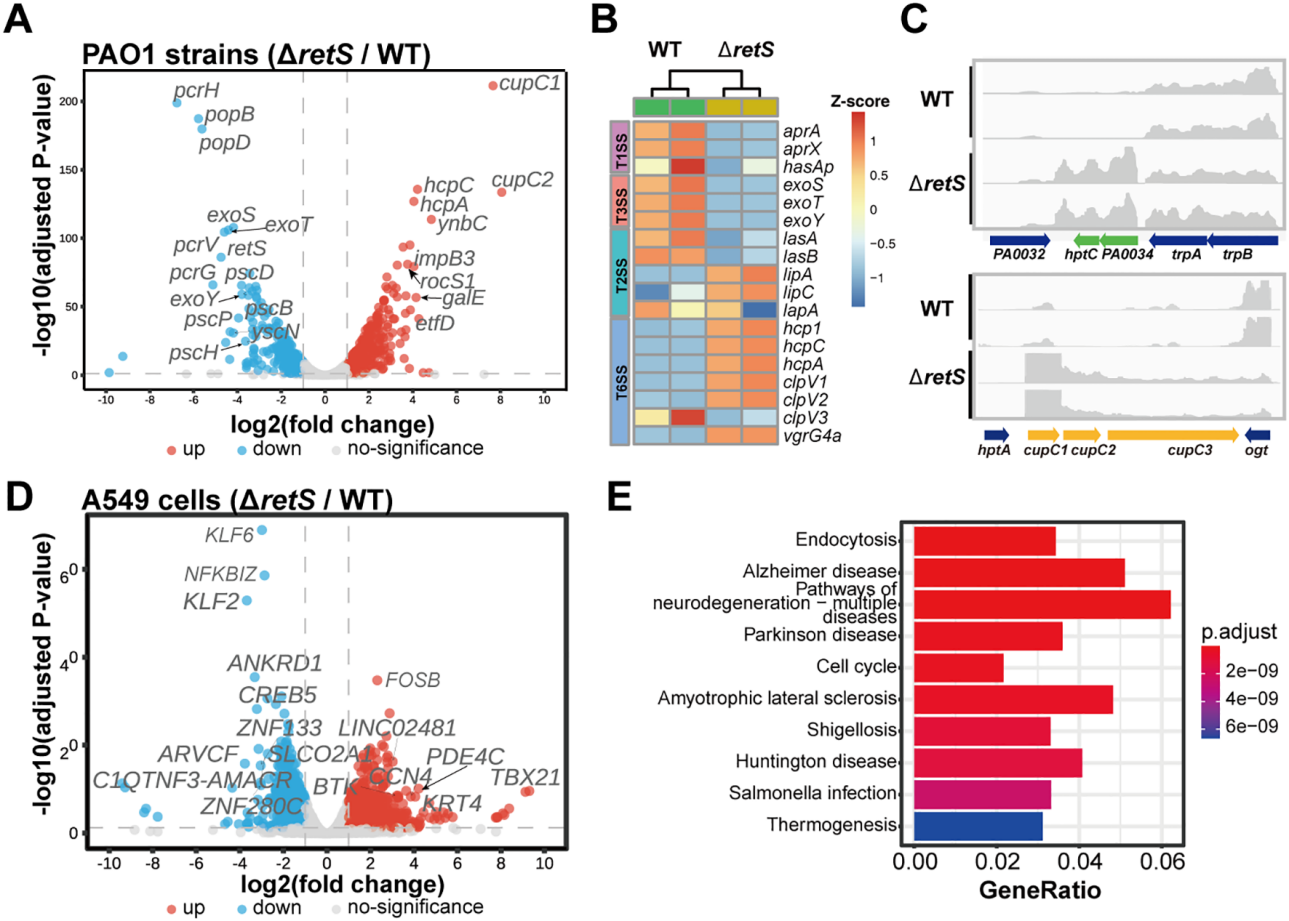
Transcriptomic analyses. **(A)** A549 cells infected with *retS* mutants and PAO1 strains, shown DEGs of comparing transcriptome of *retS* mutants and PAO1 strains in volcano plots. Dashed grey lines indicate the cutoffs (−log10[adjusted P value] > -log10(0.05), |log2[fold change]| > 1) for identifying differentially expressed genes. The upregulated (red dots) and downregulated (blue dots) genes are color-coded differently. Grey dots indicate genes with no-significant difference in transcription. **(B)** Heatmap shown the relative expression level of secretion systems genes. **(C)** RNA-seq reads aligned to the *hptC*-*PA0034* locus and *cupC* locus respectively. **(D)** A549 cells infected with *retS* mutants and PAO1, shown DEGs of A549 cells in volcano plots. **(E)** KEGG pathway enrichment of DGEs of A549 cells.

The transcriptomic results revealed a total of 373 up- and 361 down-regulated differential expression genes (DEGs) by comparing the *retS* mutant with the PAO1 WT strain (**Figure 3A**; **Supplementary Table S3**). Focusing on the secretion systems genes, we found that the expression of acute infection-related genes, such as *aprA*, *aprX* and *hasAp* of T1SS, and *exoS*, *exoT* and *exoY* of T3SS, were significantly reduced in the Δ*retS* infection group. In contrast, the expression of genes associated with the T6SS was significantly increased. Specifically, genes such as *hcp1*, *hcpC*, *hcpA*, *clpV1*, *clpV2*, *clpV3*, and *vgrG4a* exhibited marked up-regulation in the Δ*retS* infection group (**Figures 3A, B**), which is consistent with our previous experimental results (**Figure 2A**).

Interestingly, *cup* genes, particularly *cupC1*, *cupC2*, *cupB1* and *cupB2* were also found to be significantly upregulated (~203, ~267, ~7, ~2-fold, respectively) (**Figures 3A, C**; **Supplementary Table S3**). In *P. aeruginosa*, three distinct fimbrial gene loci are scattered across the chromosome. These loci include the CupA gene cluster (*PA2128*-*PA2132*), comprising five genes; the CupB gene cluster (*PA4086*-*PA4081*), consisting of six genes; and the CupC gene cluster (*PA0992*-*PA0994*), containing three genes. Taking the CupC gene cluster as an example, it encodes CupC1 as the major fimbrial subunit (MFS), CupC2 as the pilus assembly chaperone, and CupC3 as the usher protein. These gene clusters encode components for fimbrial structures *via* the chaperone-usher pathway and are essential for its pathogenicity, surface attachment and biofilm formation (Soto and Hultgren, 1999; Ruer et al., 2007). qRT-PCR analysis revealed that the expression levels of *clpV1* and *clpV2* were upregulated in the *retS* mutant-infected group, whereas *clpV3* exhibited no significant change. *cupC1* was upregulated in *retS* mutant. These findings were consistent with the RNA-seq data (**Supplementary Figure S2B**).

Recently, it has been reported that the two-component system LadS/PA0034 regulates the expression of *cupA1* (Guo et al., 2024). LadS, a calcium-responsive kinase in *P. aeruginosa*, plays a pivotal role in modulating the transition from acute to chronic infection. It governs bacterial virulence factors and biofilm formation by regulating the expression of small RNAs, including RsmY and RsmZ. PA0034 is annotated as a putative transcriptional regulator in the UniProtKB database and is likely to form a two-component system with its adjacent gene PA0033 (HptC) to regulate gene expression. We analyzed the homologous proteins of PA0034 by protein sequence comparison and found that PA0034 is highly homologous to *rocA1* and *rocA2* (**Supplementary Figure S3**).

This finding suggests that the phosphorylation relay within this two-component system may modulate the expression of genes encoding Cup fimbriae. In our study, we observed that *PA0034* and *hptC* were significantly up-regulated in the *retS* mutant (**Figure 3C**). In *P. aeruginosa*, several histidine-containing phosphotransfer proteins (Hpt) are integral to the regulation of various cellular processes *via* multistep phosphorelay systems. Notably, both *hptB* and the *hptA* genes, located immediately upstream of the *cupC* locus, did not show significant changes in expression (**Supplementary Figure S2A**). This suggests that HptC/PA0034 may be involved in the transcriptional regulation of the *cupC* gene, whereas HptA and HptB are not.

In *P. aeruginosa*, several histidine-containing phosphotransfer proteins (Hpt) are involved in the regulation of various cellular processes *via* multi-step phosphorelay systems. Notably, both the *hptB* and *hptA* genes, located immediately upstream of the *cupC* locus, did not show significant changes in expression (**Supplementary Figure S2A**). This suggests that HptC/PA0034 may be involved in the transcriptional regulation of the *cupC* gene, whereas HptA and HptB are not.

Secondly, the transcriptional responses of A549 cells infected with *retS* mutants and PAO1 strains were analyzed, and the differentially expressed genes (DEGs) were identified and visualized using volcano plots (**Figure 3D**). The results revealed that a total of 875 genes were upregulated and 1,173 genes were downregulated (**Supplementary Table S4**). The KEGG-enriched biological processes of DEGs include endocytosis, Alzheimer's disease, neurodegeneration -multiple diseases, Parkinson's disease, cell cycle, amyotrophic lateral sclerosis, shigellosis, Huntington's disease, Salmonella infection, and thermogenesis (**Figure 3E**). GSEA enrichment score curves of representative GO terms, including Apoptosis, Cell adhesion molecules, Leukocyte transendothelial migration, MAPK signaling pathway, NF-kappa B signaling pathway, PI3K/Akt signaling pathway (**Supplementary Figure S5**). Among these differentially expressed genes, FOSB, CHRM1, ENDOU, PDE4C, and EPHA8 exhibited increased expression levels in cells infected with the Δ*retS* strain. This may indicate that the cells have undergone changes or responses in some biological processes, which may involve mechanisms of cell proliferation, differentiation, migration, signal transduction, and cancer development. The expression of genes such as KLF6, KLF2, NFKBIZ, ANKRD1, and CREB5 was reduced (**Figure 3D**). KLF6 is a known tumor suppressor involved in key processes such as cell cycle regulation, apoptosis, and differentiation. KLF2 plays an important role in regulating apoptosis and inflammatory responses, and its down-regulation may lead to enhanced cellular responses to inflammatory stimuli. Down-regulation of NFKBIZ may lead to activation of the NF-κB signaling pathway, thereby promoting inflammatory responses and cell survival. Downregulated inhibitory genes may lead to enhanced cellular responses to external stimuli. Specifically, the transcriptional level of PDE4C (phosphodiesterase 4C) exhibited a log_2_[fold change] value of 4.22. PDE4C is a member of the phosphodiesterase family, which is responsible for degrading cyclic AMP (cAMP) to 5’-AMP (Wang et al., 2024). Those results revealed distinct transcriptional profiles between the *retS* mutants and the PAO1 WT strain, highlighting the regulatory impact of the *retS* gene on gene expression in the context of A549 cell infection.

### 3.4 *cupC* promotes T6SS virulence through enhancing *P. aeruginosa* attachment to host cells

Based on the observed concurrent up-regulation of the *cupC* gene cluster and the T6SS genes in our data, we hypothesize that *cupC* enhances the cytotoxic effect of the T6SS by promoting bacterial adhesion to the host cell surface, thereby facilitating T6SS function. *P. aeruginosa* harbors three *clpV* genes that encode AAA^+^ ATPases, which provide energy for the H1-, H2-, and H3-T6SS respectively. RNA-seq analysis revealed that the expression levels of *clpV3* and the structural genes of the H3-T6SS were not significantly altered, whereas the expression of genes associated with the H1- and H2-T6SS were significantly upregulated (**Supplementary Table S1**). To further investigate the roles of these T6SS systems, we constructed a Δ*retS*Δ*clpV1*Δ*clpV2* mutant (hereinafter abbreviated as Δ*retS*Δ*clpV12*) to generate a strain deficient in H1- and H2-T6SS. Additionally, we constructed a Δ*retS*Δ*cupC1*-*cupC3* mutant strain (Δ*retS*Δ*cupC*) and Δ*retS*Δ*hptC*-*PA0034* to elucidate the possible role of the *hptC*-*PA0034* operon.

**Figure 4:**
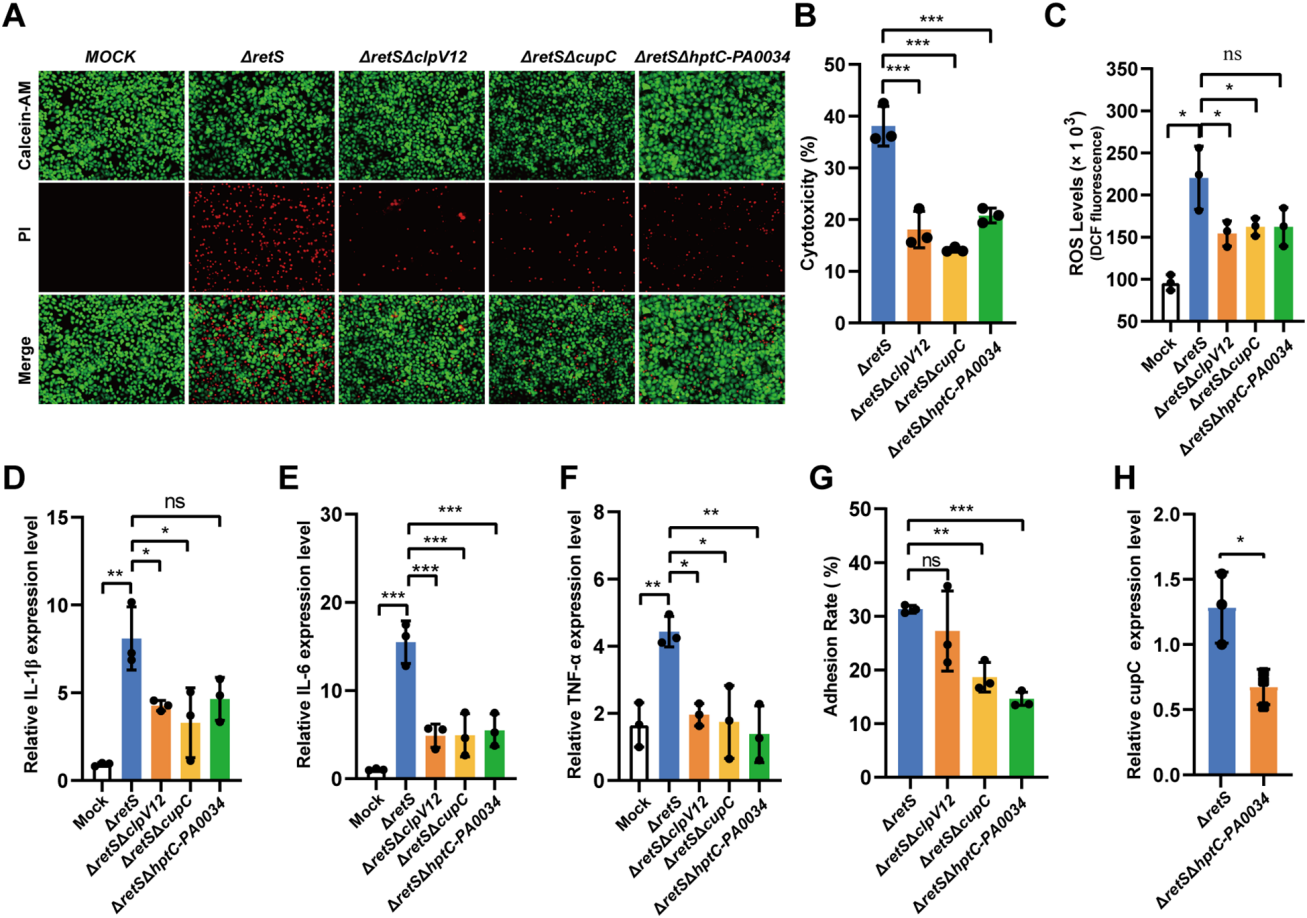
*CupC* contributes to T6SS virulence through enhancing *P. aeruginosa* attachment to host cells. **(A)** Calcein-AM/PI staining of A549 cells infected with *P. aeruginosa* strains at MOI 50 for 6 h. **(B)** LDH release assay. A549 cells were infected with *P. aeruginosa* strains at MOI 50 for 6 h and the relative levels of LDH in the co-culture supernatant were measured. **(C)** A549 cells were infected with different strains of PAs at an MOI of 50 for 7 h, and the ROS levels released by the cells were detected. **(D-F)** A549 cells infected with MOI 10 of *P. aeruginosa* strains for 12 h. The expression of inflammatory cytokines (IL-1β, IL-6, and TNF-α) were detected using qRT-PCR (compared with the uninfected group). **(G)** *P. aeruginosa* strains adhesion to A549 cells. **(H)** The effect *hptC*-*PA0034* gene mutation on expression of *cupC1* gene. The values represent the means of at least three independent experiments ± SD. ns, not significant. *P < 0.05; **P < 0.01; ***P < 0.001. n=3. Δ*retS*Δ*clpV12*, Δ*retS*Δ*clpV1*Δ*clpV2*. Δ*retS*Δ*hptC*-*PA0034*, Δ*retS*Δ*hptC*Δ*PA0034*.

First, the cytotoxicity of the Δ*retS*ΔclpV12 and Δ*retS*Δ*cupC* strains was significantly reduced compared to that of the Δ*retS* strain, as indicated by calcein-AM/PI staining and LDH cytotoxicity assays. Similarly, the Δ*retS*Δ*hptC-PA0034* strain exhibited significantly reduced cytotoxicity (P < 0.001) (**Figures 4A, B**). Second, the expression levels of inflammatory cytokines IL-1β, IL-6, and TNF-α, as well as the release of ROS, were significantly decreased in the Δ*retS*Δ*clpV12*, Δ*retS*Δ*cupC*, and Δ*retS*Δ*hptC-PA0034* strains compared to the Δ*retS* strain (**Figures 4C-F**). Third, the adhesion ability of different strains to the A549 epithelial cell line was compared. The Δ*retS*Δ*clpV12* strain exhibited no significant difference in adhesion compared to the Δ*retS* strain. In contrast, mutations in *cupC*, *hptC*, and *PA0034* each resulted in a significant reduction in bacterial adhesion to A549 cells (**Figure 4G**). Furthermore, qRT-PCR analysis revealed that deletion of *hptC*-*PA0034* led to down-regulation of *cupC1* expression (**Figure 4H**), indicating that HptC/PA0034 may positively regulate the transcription of *cupC*. These findings indicate that the cytotoxicity of the T6SS towards host cells is mediated by the *cupC* gene cluster through enhanced bacterial attachment to host cells.

### 3.5 T6SS promotes cell death, inflammatory cytokine expression, and ROS production by activating PDE4C

**Figure 5:**
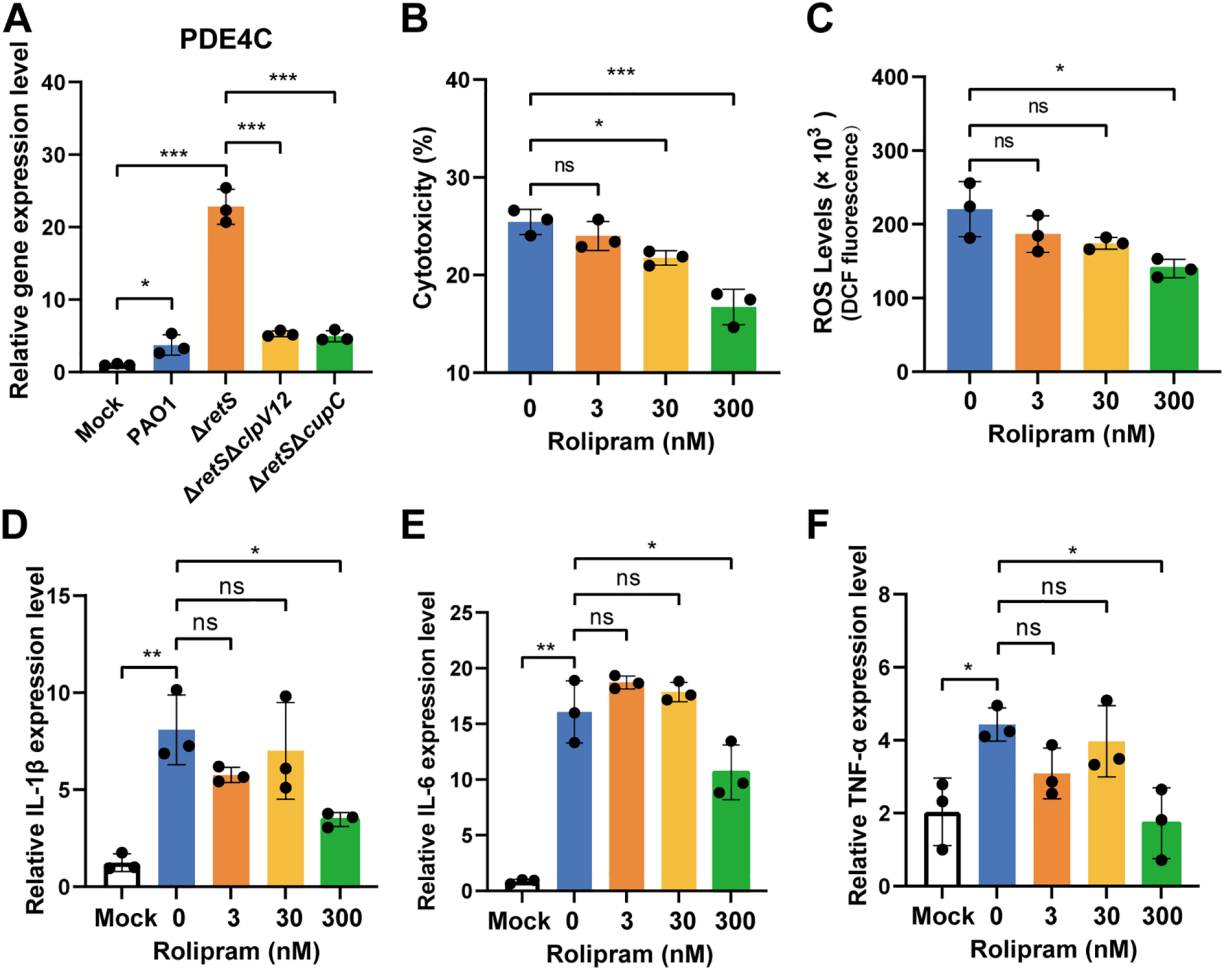
T6SS activates PDE4C to promote cell death, inflammatory cytokine expression, and ROS production. **(A)** A549 cells infected with MOI 10 of *P. aeruginosa* strains for 12 h. The expression of PDE4C was detected using qRT-PCR (compared with the uninfected group). **(B)** The LDH release experiment was used to detect the effect of T6SS on cells after adding different concentrations of Rolipram to inhibit PDE4C. **(C)** The effect of T6SS on the release of cellular ROS was detected after adding different concentrations of Rolipram to inhibit PDE4C. **(D-F)** qRT-PCR was used to detect the effect of T6SS on the expression of cellular inflammatory cytokines after different concentrations of Rolipram were added to inhibit PDE4C. The values represent the means of at least three independent experiments ± SD. ns, not significant; *P < 0.05; **P < 0.01; ***P < 0.001. n=3.

PDE4 has been reported to be involved in the pathophysiology of many inflammatory diseases, such as rheumatoid arthritis, COPD, and asthma (Kumar et al., 2024). Given these findings, we speculated that the elevated expression of PDE4C observed in the transcriptomic analysis might be attributable to the activity of the *P. aeruginosa* PAO1 T6SS. To validate this hypothesis, we compared the effects of a strain with T3SS inhibition and T6SS activation (Δ*retS*) and a strain with concurrent inhibition of both T3SS and T6SS systems (Δ*retS*Δ*clpV12*) on the expression of PDE4C in A549 cells. We found that the expression of PDE4C was significantly reduced in cells infected with the Δ*retS*Δ*clpV12* and Δ*retS*Δ*cupC* strain compared to those infected with the Δ*retS* strain for 12 h post-infection (**Figure 5A**). In addition, T6SS did not affect the expression of PDE4A, PDE4B, or PDE4D (**Supplementary Figure S4C**). Interestingly, we found that cAMP levels were significantly higher in cells infected with T6SS-deleted bacteria compared to those infected with Δ*retS* bacteria (**Supplementary Figure S4A**). The results suggest that PDE4C may serve as a host-specific molecular marker in response to T6SS activity, affecting cellular cAMP levels and thereby modulating downstream gene expression. This finding is consistent with the notion that T6SS can deliver effectors to target specific host molecules, thereby modulating host cell responses.

To further confirm the notion that PDE4 is a functional target following *P. aeruginosa* infection, we then set out to analyze the effect of PDE4 on the inflammatory response of A549 cells and the expression of cytokines. Rolipram exhibits inhibitory effects on four PDE4 subtypes, namely PDE4A, PDE4B, PDE4C, and PDE4D, with respective IC50 values of 1 nM, 1 nM, 300 nM, and 60 nM (Wang et al., 1997). First, we evaluated the cytotoxicity of Rolipram at concentrations of 3 nM, 30 nM, and 300 nM on A549 cells. Results indicated that none of these concentrations exhibited cytotoxic effects on cells (**Supplementary Figure S4B**). Subsequently, the results demonstrated that 3 nM Rolipram did not inhibit the cytotoxicity of Δ*retS* on A549 cells, whereas 30 nM and 300 nM Rolipram significantly attenuated the cytotoxic effects of Δ*retS* to different extents (**Figure 5B**). We observed that when Rolipram concentration reached 300 nM, the expression of cellular inflammatory cytokines, including IL-1β, IL-6, and TNF-α, was significantly reduced, and the release of cellular ROS was also reduced (**Figures 5C-F**). These results indicate that *P. aeruginosa* T6SS affects cellular immune damage by targeting PDE4C in A549 cells.

## 4 Discussion

*P. aeruginosa* is a major opportunistic pathogen implicated in both acute and chronic pulmonary infections, especially in immunocompromised patients and individuals with cystic fibrosis. Among its various virulence strategies, the T6SS has gained increasing attention due to its role in persistent infections. Unlike the T3SS, which mediates acute cytotoxicity through the injection of effectors into host cells, T6SS is typically activated under chronic infection conditions and functions as a contact-dependent nanomachine capable of targeting both bacterial competitors and host cells (Lertpiriyapong et al., 2012; Chen et al., 2019). In this study, by analyzing clinical isolates and using a *retS* knockout mutant, we systematically demonstrated that T6SS activation is a hallmark of *P. aeruginosa* infection and revealed novel insights into its host-regulatory mechanisms, particularly its regulation of the cAMP-specific phosphodiesterase PDE4C and the contribution of CupC fimbriae to T6SS-dependent virulence.

The transcriptomic profiling and functional assays confirmed that deletion of *retS* leads to a significant up-regulation of key T6SS genes (*hcp1*, *clpV1*, *clpV2*, *hcpC*) and a concomitant down-regulation of T3SS-related genes (*exoS*, *exoT*, *exoY*), consistent with previous findings that RetS represses T6SS *via* the GacS/A-RsmY/Z pathway (Ryan Kaler et al., 2021). Clinical strain analysis also substantiated this regulatory switch, revealing that isolates from chronic infections preferentially express higher levels of the T6SS gene *clpV2* and lower levels of the T3SS gene *exsA* compared to those from acute infections. It highlights the distinct virulence strategy employed by *P. aeruginosa* during long-term colonization of host tissues, wherein cytotoxicity is suppressed, and mechanisms promoting persistence, immune modulation, and inter-bacterial competition are favored.

The transcriptomic analysis also revealed that, compared to the PAO1 WT strain, the expression levels of the H1- and H2-T6SS genes were significantly upregulated in the *retS* mutant, consistent with previous reports (Hachani et al., 2011). In contrast, the expression of the H3-T6SS genes did not show similar changes. This discrepancy may be explained by the distinct regulatory mechanisms that govern the three T6SS clusters in *P. aeruginosa*. The H1-T6SS is primarily regulated by the GacS/A-RsmY/Z pathway, where RetS inhibits GacS activity and reduces the expression of small RNAs RsmY and RsmZ. These sRNAs normally block the translational repressor RsmA, allowing T6SS genes to be expressed (Janssen et al., 2018). The H2-T6SS is also mainly controlled by the same regulatory system and is similarly upregulated in the absence of RetS (Hachani et al., 2011). However, the regulation of H3-T6SS appears to be more intricate and influenced by additional factors beyond the GacS/A-RsmY/Z pathway. The alternative sigma factor RpoN coordinates the T6SS systems by activating the H2-T6SS but repressing the H1- and H3-T6SS (Allsopp et al., 2022). Interestingly, RpoN also exhibits divergent effects on the H3-T6SS operons (Sana et al., 2013). The quorum sensing (QS), particularly the Las and Rhl systems, have also been shown to play regulatory roles in the expression of H3-T6SS gene cluster (Sana et al., 2013; Chen et al., 2020). Furthermore, the transcriptional regulator AmrZ has been identified as an activator of H3-T6SS, promoting the expression of *vgrG3* gene (Allsopp et al., 2017). Environmental conditions also play a crucial role in modulating H3-T6SS expression. For instance, phosphate limitation has been reported to induce the expression of H3-T6SS genes through the PhoB regulatory system (Zhao et al., 2023). These studies indicate complex regulatory pathways involved in the activation of H3-T6SS. Therefore, the lack of significant up-regulation of H3-T6SS in the *retS* mutant under our experimental conditions may be due to the absence of specific environmental stimuli or regulatory signals required for its activation. This underscores the complexity of T6SS regulation in *P. aeruginosa* and highlights the need for further studies to elucidate the precise mechanisms modulating H3-T6SS.

A particularly novel finding of our study is the identification of CupC fimbriae as essential mediators of T6SS-dependent cytotoxicity. The *cupC1*-*cupC3* gene cluster, which encodes components of the chaperone-usher pathway, was significantly upregulated in the *retS* mutant. Functional deletion of the *cupC* locus not only impaired bacterial adhesion to A549 epithelial cells but also attenuated T6SS-mediated cell death, inflammatory cytokine expression (IL-1β, IL-6, TNF-α), and ROS production. These observations support the hypothesis that CupC fimbriae facilitate the physical contact required for T6SS-mediated delivery of toxic effectors into host cells (Wang et al., 2022; Liu et al., 2023). Our findings provide the first experimental evidence linking CupC fimbriae to T6SS virulence, expanding the functional repertoire of bacterial adhesins beyond traditional biofilm formation and colonization roles.

Mechanistically, we also explored the upstream regulatory signals that may modulate *cupC* expression in the context of T6SS activation. Transcriptome data revealed significant up-regulation of the *hptC-PA0034* operon. PA0034 shares structural features with RocA family regulators, which are known to control Cup gene expression through the Roc two-component systems, Roc1 and Roc2 (Giraud et al., 2011). The key components of Roc system include the histidine kinases RocS1 and RocS2, and the response regulators RocA1, RocR and RocA2 (Sivaneson et al., 2011). In this study, we observed that the genes *rocA1*, *rocA2*, *rocS1*, *rocS2*, and *rocR* were significantly upregulated in the *retS* mutant (**Supplementary Table S3**). Deletion of the *hptC*-*PA0034* operon resulted in down-regulation of the *cupC1* gene (**Figure 4H**) and diminished the adhesion ratio and virulence on A549 cell. Additionally, this *hptC*-*PA0034* mutation reduced the levels of pro-inflammatory cytokines and ROS. These findings suggest that *P. aeruginosa* possesses a complex signaling network involving both the Roc and HptC/PA0034 pathways to regulate fimbrial gene expression in response to environmental cues or during host infection.

Abnormal expression of PDE4 is associated with a variety of diseases, including asthma, COPD, depression, and cardiovascular disease (Ferrari et al., 2016; Jin et al., 2023; Puertas-Umbert et al., 2023; Nahid et al., 2025). By regulating cAMP levels, PDE4 influences the activation of protein kinase A (PKA) and EPAC (exchange protein directly activated by cAMP), both of which mediate downstream signaling pathways (Tiwari et al., 2004; Golkowski et al., 2016). A markedly up-regulation of the PDE4C gene was found in A549 cells following infection with the *retS* mutant (**Supplementary Table S4**). However, PDE4A, PDE4B, and PDE4D expression remained unchanged (**Supplementary Figure S4D**), indicating a specific effect of T6SS activity on PDE4C induction. Functional assays further confirmed this relationship: deletion of T6SS genes (*clpV1, clpV2*) or *cupC* abolished PDE4C up-regulation and reduced the levels of pro-inflammatory cytokines and ROS. Moreover, pharmacological inhibition of PDE4 activity using Rolipram alleviated inflammation and cytotoxicity. These findings suggest that T6SS-mediated up-regulation of PDE4C may represent a key host-pathogen interaction axis that promotes inflammation and tissue damage. It raises the possibility that PDE4C could serve as a biomarker of T6SS-mediated host response or even as a therapeutic target in chronic *P. aeruginosa* infections. The molecular mechanisms by which T6SS regulates PDE4C expression and its downstream signaling network remain to be elucidated.

Given the extensive repertoire of T6SS effectors in *P. aeruginosa*, future studies should focus on identifying specific effectors that contribute to the pathogenicity of *P. aeruginosa* in chronic infections. This approach may facilitate the development of strategies to block the virulence of *P. aeruginosa*.

## 5 Conclusion

**Figure 6:**
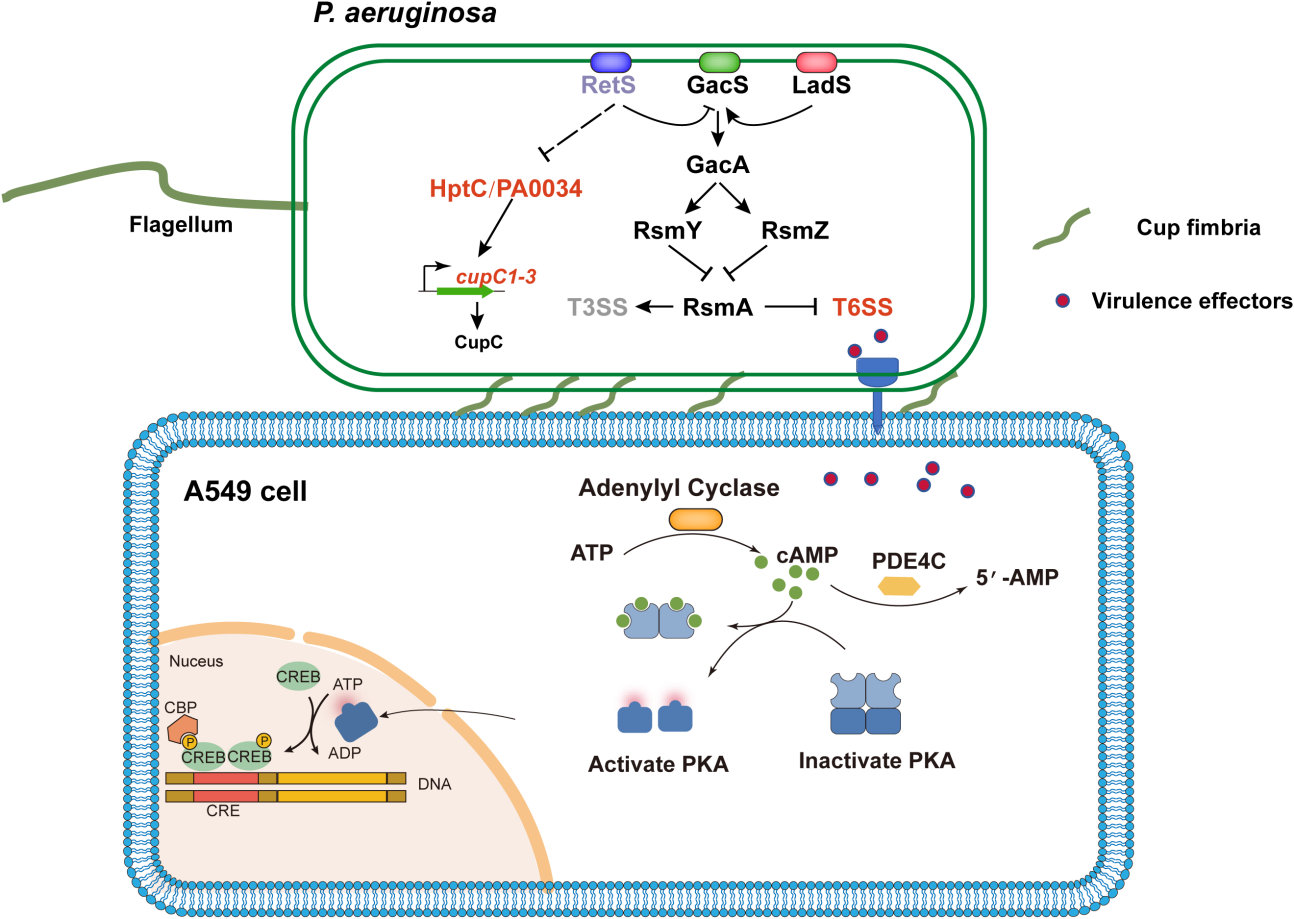
Schematic diagram of *P. aeruginosa* Cup fimbriae-mediated T6SS virulence activation of host PDE4C leading to cell damage. The RetS-GacS/GacA regulatory pathway controls the switch on/off T6SS and T3SS by modulating the levels of small RNAs (RsmZ and RsmY) that sequester the translation repressor RsmA. Subsequently, the effectors of T6SS thereby activating the PDE4C pathway in host cell A549. The PDE4C catalyzes the conversion of the secondary messenger molecule cAMP to AMP, thereby modulating the PKA. This process partially elicits the production of inflammatory cytokines, ROS, and other cellular responses, ultimately culminating in cellular damage. Concurrently, deletion of the *retS* gene alleviates the repression of *hptC*-*PA0034* operon expression, thereby enhancing the transcription of the *cupC* operon. The up-regulation of the *cupC* operon promotes the assembly of Cup fimbriae, which mediate bacterial adhesion to host cells and facilitate the contact-dependent delivery of T6SS effectors. The lines indicate the interactive relationship: Arrow, activation; T bar, repression; Dashed line indicates a predicted regulatory role.

Our study reveals a novel virulence mechanism of *P. aeruginosa*, in which CupC-mediated adhesion facilitates close contact with host epithelial cells, enhancing T6SS-mediated effector delivery. Using a T6SS-hyperactive strain (Δ*retS*) to infect A549 cells, combined with transcriptomic analyses, we demonstrate that the *cupC* operon promotes bacterial adhesion and cytotoxicity, while also inducing host PDE4C expression and disrupting the cAMP pathway. Importantly, our findings focus specifically on the roles of the H1- and H2-T6SS, which are upregulated in the Δ*retS* mutant and are primarily responsible for the observed phenotypes. These results highlight a cooperative role of CupC and T6SS in mediating host cell injury and inflammatory responses, providing new insights into *P. aeruginosa* pathogenesis and suggesting potential therapeutic targets for chronic *P. aeruginosa* infections (**Figure 6**).

## Data Availability

The original contributions presented in the study are publicly available. This data can be found here: [https://www.ncbi.nlm.nih.gov/bioproject?term=PRJNA1204113].

## Supplementary material

Supplementary Figure 1 Construction of gene knockout strains based on PAO1. **(A)**, Sanger sequencing results of the deletion of the *retS*, *cupC(cupC1-cupC3)*, *clpV1, clpV2 and PA0033–34* genes *via* homologous recombination. The length of the knockout sequence is indicated. The red arrow indicates the upstream sequence, and the black arrow indicates the downstream sequence from the knockout site. The nucleotide sequence is presented with the 5’-end on the left and the 3’-end on the right by default. **(B)**, The growth curves of the Δ*retS*, Δ*retS*Δ*cupC*, Δ*retS*Δ*clpV1*Δ*clpV2* (Δ*retS*Δ*clpV12*), and Δ*retS*Δ*hptC*-*PA0034* mutant strains were obtained in LB medium. **(C)**, Bacterial cell killing assay. The *retS* mutant exhibited enhanced competitive ability compared with PAO1 WT strain. **(D)**, The *retS* mutation increased the biofilm formation of *P. aeruginosa* as assessed by crystal violet staining. **(E)**, Pyocyanin production of *P. aeruginosa* strains. Values were expressed as the means of at least three independent experiments. **P* < 0.05, ***P*  <  0.01.

Supplementary Figure 2 Visualization and validation of transcriptomic Data. **(A)**, Visualization analysis of RNA-seq reads mapping on PAO1 genome using IGV software. Reads coverage of the *hptA*, *hptB* and *hptC* locus of PAO1 and *retS* mutant in A549 cell infection condition. WT, wide-type of PAO1 strain. *retS*, PAO1 *retS* gene mutant. The red color indicates the coverage of reads aligned to the positive strand, while the blue color indicates the coverage of aligned reads to the negative strand. **(B)**, qRT-PCR validation of the RNA-seq results. *P. aeruginosa* strains infected of A549 cells under the same experimental condition with RNA-seq assay. qRT-PCR analysis revealed that the expression levels of *clpV1* and *clpV2* were upregulated in the *retS* mutant-infected group, whereas *clpV3* exhibited no significant change. *cupC1* was upregulated in *retS* mutant. ns, *P* > 0.05; ***P*  <  0.01; ****P*  <  0.001; n=3.

Supplementary Figure 3 Proteins sequence alignment. **(A, B)**, The protein alignment of PA0991 (HptA), PA3345 (HptB) with PA0033 (HptC) by MAFFT FFT-NS-1 (v7.520). **(C, D)**, The protein alignment of PA3045 (RocA2) and PA3948 (RocA1) with PA0034.

Supplementary Figure 4
**(A)**, The expression of cAMP in cells was detected using ELISA Kit after 12 h of infection with PAO1 or Δ*retS* at a MOI of 10, or in uninfected cells. **(B)**, Assessment of the toxicity of different concentrations of Rolipram on A549 cells using CCK-8 assay. **(C)**, The expression levels of PDE4A, PDE4B, and PDE4D in cells were detected by qRT-PCR after infection with MOI=10 for 12 h. ns, *P* > 0.05; **P* < 0.05; ****P* < 0.001; n=3.

Supplementary Figure 5 GSEA enrichment score curves of representative GO terms, including Apoptosis, Cell adhesion molecules, Leukocyte transendothelial migration, MAPK signaling pathway, NF-kappa B signaling pathway, PI3K/Akt signaling pathway.

Supplementary Table 1 Primers used in this study: **Supplementary Table S1**.docx.

Supplementary Table 2 Bacterial strains and plasmids used in this study: **Supplementary Table S2**.docx.

Supplementary Table 3 PAO1 strains transcriptome data: **Supplementary Table S3**.xlsx.

Supplementary Table 4 A549 cells transcriptome data: **Supplementary Table S4**.xlsx.
