# Supplementary figures and images for: Transcriptomic profiling reveals RetS-mediated regulation of type VI secretion system and host cell responses in Pseudomonas aeruginosa infections
Source: Front Cell Infect Microbiol. 2025 Jun 10;15:1582339. doi: 10.3389/fcimb.2025.1582339 (PMC12185982; doi:10.3389/fcimb.2025.1582339)

### Supplementary Figure 1

**A**

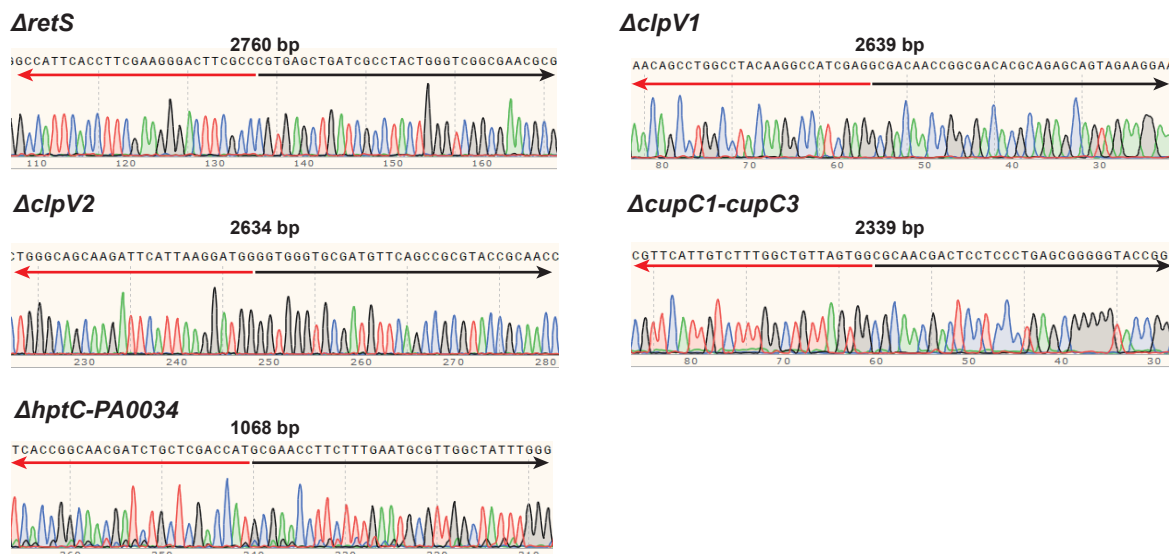

**B**

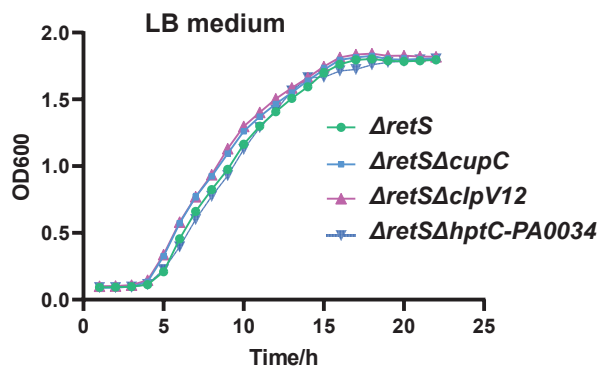

**C**

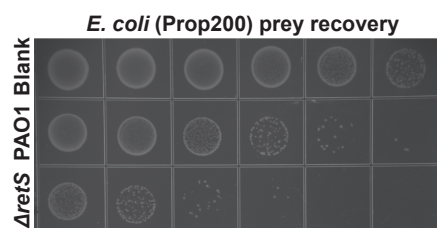

D

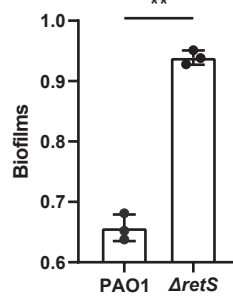

**E**

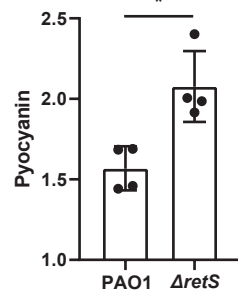

Supplement: Supplementary Figure 1 — Construction of gene knockout strains based on PAO1. (A), Sanger sequencing results of the deletion of the retS, cupC(cupC1-cupC3), clpV1, clpV2 and PA0033–34 genes via homologous recombination. The length of the knockout sequence is indicated. The red arrow indicates the upstream sequence, and the black arrow indicates the downstream sequence from the knockout site. The nucleotide sequence is presented with the 5’-end on the left and the 3’-end on the right by default. (B), The growth curves of the ΔretS, ΔretSΔcupC, ΔretSΔclpV1ΔclpV2 (ΔretSΔclpV12), and ΔretSΔhptC-PA0034 mutant strains were obtained in LB medium. (C), Bacterial cell killing assay. The retS mutant exhibited enhanced competitive ability compared with PAO1 WT strain. (D), The retS mutation increased the biofilm formation of P. aeruginosa as assessed by crystal violet staining. (E), Pyocyanin production of P. aeruginosa strains. Values were expressed as the means of at least three independent experiments. *P < 0.05, **P < 0.01. [file DataSheet1.pdf]

Supplementary Figure 2

A

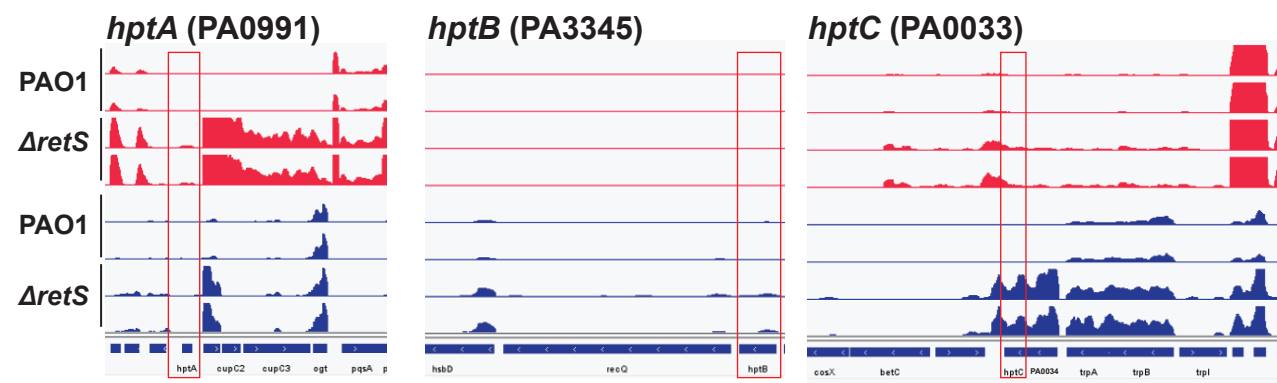

B

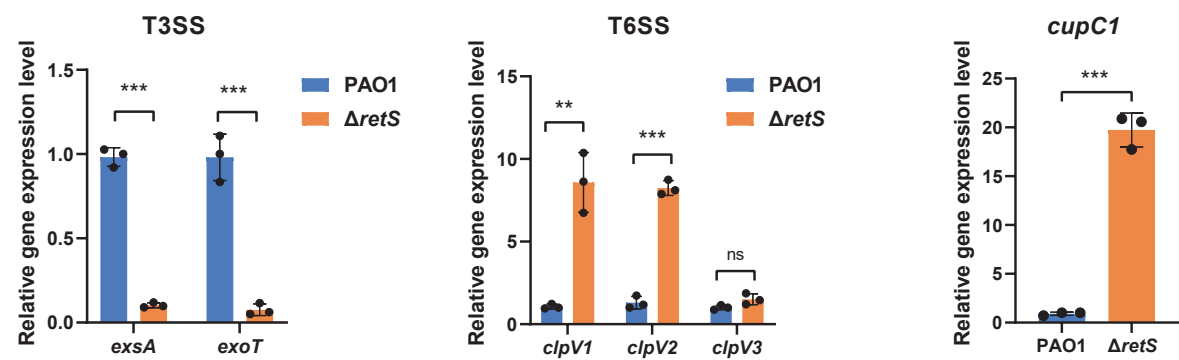

Supplement: Supplementary Figure 2 — Visualization and validation of transcriptomic Data. (A), Visualization analysis of RNA-seq reads mapping on PAO1 genome using IGV software. Reads coverage of the hptA, hptB and hptC locus of PAO1 and retS mutant in A549 cell infection condition. WT, wide-type of PAO1 strain. retS, PAO1 retS gene mutant. The red color indicates the coverage of reads aligned to the positive strand, while the blue color indicates the coverage of aligned reads to the negative strand. (B), qRT-PCR validation of the RNA-seq results. P. aeruginosa strains infected of A549 cells under the same experimental condition with RNA-seq assay. qRT-PCR analysis revealed that the expression levels of clpV1 and clpV2 were upregulated in the retS mutant-infected group, whereas clpV3 exhibited no significant change. cupC1 was upregulated in retS mutant. ns, P > 0.05; **P < 0.01; ***P < 0.001; n=3. [file DataSheet2.pdf]

Supplementary Figure 4

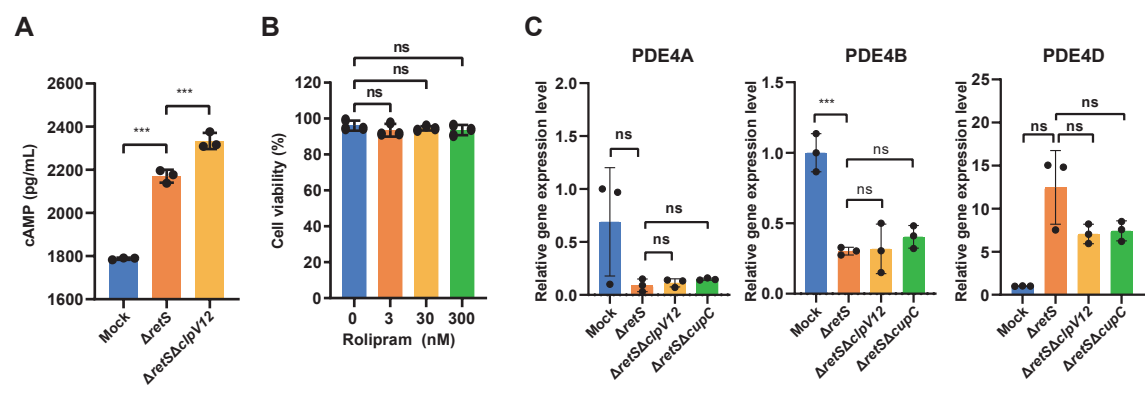

Supplement: Supplementary Figure 4 — (A), The expression of cAMP in cells was detected using ELISA Kit after 12 h of infection with PAO1 or ΔretS at a MOI of 10, or in uninfected cells. (B), Assessment of the toxicity of different concentrations of Rolipram on A549 cells using CCK-8 assay. (C), The expression levels of PDE4A, PDE4B, and PDE4D in cells were detected by qRT-PCR after infection with MOI=10 for 12 h. ns, P > 0.05; *P < 0.05; ***P < 0.001; n=3. [file DataSheet4.pdf]

gseaplot2

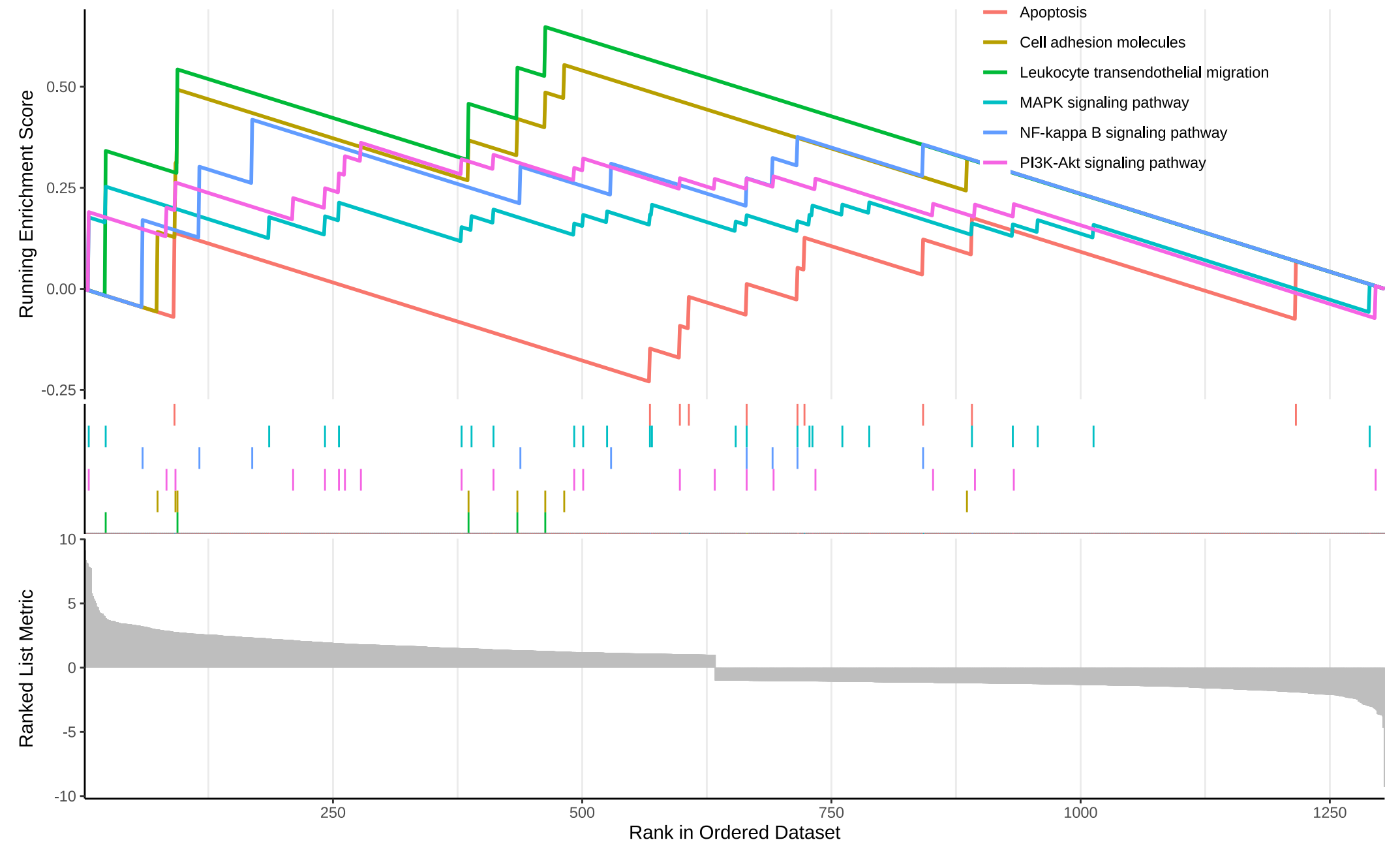

Supplement: Supplementary Figure 5 — GSEA enrichment score curves of representative GO terms, including Apoptosis, Cell adhesion molecules, Leukocyte transendothelial migration, MAPK signaling pathway, NF-kappa B signaling pathway, PI3K/Akt signaling pathway. [file DataSheet5.pdf]
