# Supplementary material for: Transcriptomic profiling reveals RetS-mediated regulation of type VI secretion system and host cell responses in Pseudomonas aeruginosa infections
Source: Front Cell Infect Microbiol. 2025 Jun 10;15:1582339. doi: 10.3389/fcimb.2025.1582339 (PMC12185982; doi:10.3389/fcimb.2025.1582339)
Supplement: Supplementary Figure 3 — Proteins sequence alignment. (A, B), The protein alignment of PA0991 (HptA), PA3345 (HptB) with PA0033 (HptC) by MAFFT FFT-NS-1 (v7.520). (C, D), The protein alignment of PA3045 (RocA2) and PA3948 (RocA1) with PA0034. [file DataSheet3.pdf]

Supplementary Figure 3

A

```
PA0033          ---------MRT-FDLTAALGDRANQPEISRLLVAQLMTSNNEDTRKLRRAQEREQTQEL
PA0991          MKELGSESPMESLFDIRSLEAITGGEPAALQRLLETLLASSRDDRTRLAELLALADLQGV
                         *.: **: :  .  ..:*   : *:  *::*..:*  :* .     : * :

PA0033          NELAHRLAGMASLIHAHAVERACHQLEIAC---GESSVR--KLADAVDDLVTALG-ELQC
PA0991          AELAHRIKGAARVVGSSLLVESCSCLEKACQNPSESSERIANLASSLEGKLSAFEISIST
                 *****: * * :: :  : .:*  ** **   .*** *  :**.:::. ::*:  .:.

PA0033          ELHDWMTMTAPQGTAKS
PA0991          EL------------AKL
                **            **
```

B

```
PA0033          MRTFDLTAA-LGDR--ANQPEISR----LLVAQLMTSNNEDTRKLRRAQEREQTQELNEL
PA3345          -----MSAPHLDDRVLASLQEVMEDEYPVLLDTFVLDSEERLRSLHAALQAGDAQALRHT
                     ::*. *.**  *.  *: .    :*:  :: ..:*  *.*: * :  ::* *..

PA0033          AHRLAGMASLIHAHAVERACHQLEIACGESSVRKLADAVDDLVTALGELQCELHDWMTMT
PA3345          AHSFKGGSSNMGAVLLAGYCKELE----ESARR-------------GELQ---------R
                ** : * :* : *  :   *::**    **: *             ****

PA0033          APQGTAKS-------------------
PA3345          APALIEQMEREFAIVRILFKQERQRYR
                **    :
```

C

```
PA0034          MSKVLIVDDHPAIRLAVRLLFERDGFTIVGEADNGAEALQVARKKSPDLAILDIGIPKID
PA3045          MSRILIVDDHPVIRMAMKMLLEAEGHQIVGDTDNGVDAISLGRELKPDLVILDIGIPRLD
                **::*******.**:*:::*:* :*. ***::***.:*:.:.*: .***.*******::*

PA0034          GLEVIARLKSLKLDTKVLVLTRQNPAQFAPRCLQAGAMGFVSKRENLSELLLAAKAVLAG
PA3045          GLEVISRLMVLALPLKILVLTGQSASLFALRSMQAGAAGFVCKQGGLAELVTAVNAVASG
                *****:**   * *  *:**** *..: ** *.:**** ***.*: .*:**: *.:** :*

PA0034          YIHFPTGALRSINQQSRDNEARMLESLSDREMTVLQYLANGNTNKAIAQQLFLSEKTVST
PA3045          YSYFPSSAMRPVQQGAYSDDVELLGRLSDREVSVLQYLSQGYSNKQISEQMFISNKTVST
                * :**:.*:*.::* : .::..:*  *****::*****::* :** *::*:*:*:*****

PA0034          YKSRIMLKLNAHSLAGLIDFARRHELI
PA3045          YKARLLLKLNAGSLVDLIEFAKRNTLI
                **:*::***** **..**:**:*: **
```

D

```
PA0034          MSKVLIVDDHPAIRLAVRLLFERDGFTIVGEADNGAEALQVARKKSPDLAILDIGIPKID
PA3948          MHTVLIVDDHPVIRLAVRVLLEKHGLQVVAETDNGVDAIQLVREHEPDVVILDIGIPKLD
                * .********.******:*:*:.*: :*.*:***.:*:*:.*::.**:.********:*

PA0034          GLEVIARLKSLKLDTKVLVLTRQNPAQFAPRCLQAGAMGFVSKRENLSELLLAAKAVLAG
PA3948          GLTVISRIKSLGLRSQVLVLTSQSAEAFCKRCIQVGARGFVNKEEDLNNLINALNAVTAG
                ** **:*:*** * ::***** *..  *. **:*.** ***.*.*:*.:*: * :** **

PA0034          YIHFPTGALRSINQ-QSRDNEARMLESLSDREMTVLQYLANGNTNKAIAQQLFLSEKTVS
PA3948          YTFFPSLTFDTLGTPPERISESELIGLLSDREMIVLQHLAMGYSNKAIGEKLFLSNKTVS
                * .**: :: ::.   .* .*:.::  ****** ***:** * :****.::****:****

PA0034          TYKSRIMLKLNAHSLAGLIDFARRHELI-
PA3948          TYKTRLLQKLGLGSLVDLAEFAKRNSLIH
                ***:*:: **.  **..* :**:*:.**
                **    :
```
