# Supplementary material for: Transcriptomic profiling reveals RetS-mediated regulation of type VI secretion system and host cell responses in Pseudomonas aeruginosa infections
Source: Front Cell Infect Microbiol. 2025 Jun 10;15:1582339. doi: 10.3389/fcimb.2025.1582339 (PMC12185982; doi:10.3389/fcimb.2025.1582339)
Supplement: Supplementary Table 1 — Primers used in this study: Supplementary Table S1 .docx. [file Table1.docx]

**Supplementary Table 1.** Primers used in this study

| **Primer** | | **Sequence (5'→3')** |
| --- | --- | --- |
| **Constructing *retS* deletion mutant** | | |
| *retS*-P1 | | acggccagtgaattcgagctccgcaacttcaccgaggtcga |
| *retS*-P2 | | ggcgatcagctcacgggcgaagtcccttcgaaggt |
| *retS*-P3 | | cgaagggacttcgcccgtgagctgatcgcctactg |
| *retS*-P4 | | ggctggatcccaagctctagagccagtgcgcagacgaacag |
| *retS*-M1 | | tgcttctaccgcaaggac |
| *retS*-M2 | | gtggcatgtgacaacga |
| **Constructing *clpV1* deletion mutant** | | |
| *clpV_1_*-P1 | | cggccagtgaattcgagctccgtccgaacctctgaacctg |
| *clpV_1_*-P2 | | tctgcgtgtcgccggttgtcgcctcgatggccttgtaggc |
| *clpV_1_*-P3 | | cctacaaggccatcgaggcgacaaccggcgacacgcagag |
| *clpV_1_*-P4 | | gctggatcccaagctctagaggcaggagacggaacgactg |
| *clpV_1_*-M1 | | cctgcaacgccacgaa |
| *clpV_1_*-M2 | | cagcgggcaatccacc |
| **Constructing *clpV2* deletion mutant** | | |
| *clpV_2_*-P1 | | acggccagtgaattcgagctccacagggagacgtgtaccaa |
| *clpV_2_*-P2 | | aacatcgcacccaccccatccttaatgaatcttgc |
| *clpV_2_*-P3 | | attcattaaggatggggtgggtgcgatgttcagcc |
| *clpV_2_*-P4 | | ggctggatcccaagctctagagatgggaaactgcgccagcc |
| *clpV_2_*-M1 | | ggcaagttccgcattcat |
| *clpV_2_*-M2 | | gatacaactggctcagct |
| **Constructing *cupC* deletion mutant** | | |
| *cupC*-P1 | | cggccagtgaattcgagctcgcagcgatggctcctcc |
| *cupC*-P2 | | ctcagggaggagtcgttgcgccactaacagccaaagacaatg |
| *cupC*-P3 | | ttgtctttggctgttagtggcgcaacgactcctccct |
| *cupC*-P4 | | gctggatcccaagctctagagttcagactcgaatcaccatg |
| *cupC*-M1 | | gctgctcaggtttctccac |
| *cupC*-M2 | | ccttcagggcacgaatgtc |
| **Constructing PA0033-34 deletion mutant** | | |
| PA0033-PA0034-P1 | | acggccagtgaattcgagctccagaccgtcatgcaccacat |
| PA0033-PA0034-P2 | | gcaacgatctgctcgaccatgcgaaccttctttgaatgcg |
| PA0033-PA0034-P3 | | cgcattcaaagaaggttcgcatggtcgagcagatcgttgc |
| PA0033-PA0034-P4 | | ggctggatcccaagctctagactctgttccagcgcgttcat |
| PA0033-PA0034-M1 | | tcggtatcggcttcggcatc |
| PA0033-PA0034-M2 | | caggacctggtggtgttcat |
| **For qRT PCR** | | |
| q-*cupC1*-F | | atggcgaaggcaatgaca |
| q-*cupC1*-R | | cagggtgaacggcaagg |
| q-*clpV1*-F | | cgtcgcaagccctacag |
| q-*clpV1*-R | | tcgccgtcctccatca |
| q-*clpV2*-F | | tttcacccacaaccttaccca |
| q-*clpV2*-R | | gcgctgccttcacttcgtc |
| q-*clpV3*-F | | gccgcaagccctattcg |
| q-*clpV3*-R | | cggtgccgtcttccatca |
| q-*hsiA2*-F | | gggtgaggatgtgcgtttt |
| q-*hsiA2*-R | | aaggattcccgttggtagag |
| q-*exsA*-F | | tcagtcctatttcacccag |
| q-*exsA*-R | | ggcattcgtccttcc |
| q-*exoT*-F | | gaggcggtgaaagaggg |
| q-*exoT*-R | | gccgaacagggtggttat |
| q-*rpoD*-F | | ctgaagatcgccaaagagcc |
| q-*rpoD*-R | | gtgtggtcggtgttcatgtc |
| q-IL1β-F | | gtgaggaggacgaacatc |
| q-IL1β-R | | gagccagaagaggttgag |
| q-IL6-F | | cacctcttcagaacgaattgac |
| q-IL6-R | | gatgattttcaccaggcaagt |
| q-TNFα-F | | cctctctctaatcagccctctg |
| q-TNFα-R | | gaggacctgggagtagatgag |
| q-PDE4A-F | | cctgggcggaccttg |
| q-PDE4A-R | | ccggatggcgctgtagta |
| q-PDE4B-F | atgtgggcatgagttggg | |
| q-PDE4B-R | caaagtcagtagttcgggagc | |
| q-PDE4C-F | ggggcaatggatggtaaa | |
| q-PDE4C-R | taaatgggtgggaaagtgaa | |
| q-PDE4D-F | cacaatccctcagagccc | |
| q-PDE4D-R | ttgactgccactgtcctttt | |
| q-*β-actin*-F | atcgtgcgtgacattaaggagaa | |
| q-*β-actin*-R | aggaaggaaggctggaagagt | |
