# Supplementary material for: Transcriptomic profiling reveals RetS-mediated regulation of type VI secretion system and host cell responses in Pseudomonas aeruginosa infections
Source: Front Cell Infect Microbiol. 2025 Jun 10;15:1582339. doi: 10.3389/fcimb.2025.1582339 (PMC12185982; doi:10.3389/fcimb.2025.1582339)
Supplement: Supplementary Table 2 — Bacterial strains and plasmids used in this study: Supplementary Table S2 .docx. [file Table2.docx]

**Supplementary Table 2.** Bacterial strains and plasmids used in this study

| **Strain or plasmid** | **Relevant characteristics** | **Source** |
| --- | --- | --- |
| ***P. aeruginosa*** | | |
| PAO1 | *Pseudomonas aeruginosa* PAO1, Wild-type prototroph | This lab |
| Δ*retS* | A mutant strain of PAO1 with deletions of the *retS* gene | This study |
| Δ*retS*Δ*clpV_12_* | A mutant strain of PAO1 with deletions of the *retS*, *clpV_1_*, and *clpV_2_* genes | This study |
| Δ*retS*Δ*cupC* | A mutant strain of PAO1 with deletions of the *retS*, *cupC1, cupC2 and cupC3* genes | This study |
| Δ*retS*Δ*hptC-PA0034* | A mutant strain of PAO1 with deletions of the *retS*, PA0033, and PA0034 genes | This study |
| **Plasmids** | | |
| pGSM | A plasmid containing the gentamicin resistance gene (GmR), the sacB gene, and the mob gene for bacterial conjugation | This lab ^[1]^ |
| pGSM-*retS* | A fragment with *retS* upstream and downstream homology arms was inserted into *SacI/XbaI* sites of pGSM | This study |
| pGSM-*clpV_1_* | A fragment with *clpV_1_* upstream and downstream homology arms was inserted into *SacI/XbaI* sites of pGSM | This study |
| pGSM-*clpV_2_* | A fragment with *clpV_2_* upstream and downstream homology arms was inserted into *SacI/XbaI* sites of pGSM | This study |
| pGSM-*cupC* | A fragment with *cupC1-cupC3* upstream and downstream homology arms was inserted into *SacI/XbaI* sites of pGSM | This study |
| pGSM-PA0033-34 | A fragment with PA0033-PA0034 upstream and downstream homology arms was inserted into *SacI/XbaI* sites of pGSM | This study |

Reference

1. Zeng, J., N. Zhang, B. Huang, R. Cai, B. Wu, S. E, C. Fang, and C. Chen, *Mechanism of azithromycin inhibition of HSL synthesis in Pseudomonas aeruginosa.* Sci Rep, 2016. **6**: p. 24299. DOI: <https://doi.org/10.1038/srep24299>.
